# Preparation of ACE Inhibitory Peptides from *Mytilus coruscus* Hydrolysate Using Uniform Design

**DOI:** 10.1155/2013/290120

**Published:** 2012-12-26

**Authors:** Jin-Chao Wu, Jie Cheng, Xiao-lai Shi

**Affiliations:** The Second Institute of Oceanography, SOA, Hangzhou 310012, China

## Abstract

The angiotensin-I-converting enzyme (ACE) inhibitory peptides from mussel, *Mytilus coruscus*, were investigated and the variable factors, protease concentration, hydrolysis time, pH, and temperature, were optimized using Uniform Design, a new statistical experimental method. The results proved that the hydrolysate of alkali proteases had high ACE-inhibitory activity, especially the alkali protease E1. Optimization by Uniform Design showed that the best hydrolysis conditions for preparation of ACE-inhibitory peptides from *Mytilus coruscus* were protease concentration of 36.0 U/mL, hydrolysis time of 2.7 hours, pH 8.2, and Temperature at 59.5°C, respectively. The verification experiments under optimum conditions showed that the ACE-inhibitory activity (91.3%) were agreed closely with the predicted activity of 90.7%. The amino acid composition analysis of *Mytilus coruscus* ACE-inhibitory peptides proved that it had high percent of lysine, leucine, glycine, aspartic acid, and glutamic acid.

## 1. Introduction

About 30% of Americans are suffering hypertension and risk of cardiovascular disease development as an independent factor [1]. Hypertension is one of the most frequent chronic diseases and the incidence of this disease was increased in recent years. This disease affected about 65% of 65–75-year-old people in Western developed countries and its incidence was increased with age [2]. Angiotensin-I-converting enzyme (EC 3.4.15.1; ACE) is one of the metalloproteases and zinc is neccessary for its activity [3]. ACE cleaves dipeptides from oligopeptide's carboxylic terminus, which plays important physiological role in blood pressure regulation [4]. Functional foods, containing ACE inhibitory peptides, may control blood pressure moderately. Many ACE inhibitory peptides *in vitro* have been isolated from various food derived proteins hydrolysate, such as milk [5], seed protein [6], blue mussel protein [7], bovine blood plasma [8], casein [9–11], zein [12], sardine [13], and tuna muscle [14]. ACE inhibitors have also been isolated from fermented foods, such as yoghourt [15], soy sauce [16], and soybean [17]. 

*Mytilus coruscus* is one of the most important bivalves in both Chinese aquaculture and Chinese market [18]. Like other marine animals, some biactive peptides have been reported from *Mytilus* mussel protein, such as *Mytilus* inhibitory peptides [19], antimicrobial peptides [20], and anticoagulant peptide [21]. In addition, an ACE inhibitory peptide has been purified by chromatography method and identified from blue mussel sauce [7]. However, there was no report to obtain ACE inhibitory peptides from *Mytilus coruscus* mussel protein hydrolysate.

Uniform Design method, a new experimental technique, is established together by Fang [22]. One of the most important advantages of the Uniform Design is that many factors and levels can be desined simulataneously. Uniform Design offers many convenient experimental tables [23]. But, unlike orthogonal design, the largest possible amount of levels for each factor can be allowed in Uniform Design, and so much so that the number of levels sometimes can be equal to the number of experiment runs [24]. As a statistical and experiment design technique, Uniform Design method has been successfully used for many experiments, especially in optimizing processes [23, 25, 26].

In the present study, we want to optimize the hydrolysis conditions for achieving ACE inhibitory peptides from *Mytilus coruscus* muscle protein. Uniform Design method was applied to investigate the effects of protease concentration, hydrolysis time, hydrolysis temperature, and hydrolysis pH for the ACE inhibitory activity of hydrolysates from *Mytilus coruscus*.

## 2. Materials and Methods: ACE from Rabbit Lung

### 2.1. Materials

Mussel, *Mytilus coruscus*, was obtained from local aquatic product market (Hangzhou, China). Hippuryl-histidyl-leucine (HHL) was used as substrate of ACE. The HHL and ACE were purchased from local chemical company (Hangzhou, China). Five kinds of proteases (E1 to E5) were purchased from local food additives market (Hangzhou, China). The labeled optimum hydrolysis temperature and pH were shown in Table 1. All other reagents were analytical grade chemicals.

### 2.2. Preparation of Hydrolysates

Mussels,* Mytilus coruscus,* were washed with water to remove salt and other materials. The mussels were filleted and defatted with petroleum ether at 50°C by reflux extraction. Then the mussels were minced and mixed with distilled water (ratio of 1 : 10). The mixture was homogenate and then was boiled for 10 minutes to inactive the inner protease. Then the mixture was digested by five proteases at designed conditions, respectively. The pH of the reaction mixture was maintained stably by addition of either 1 N NaOH or HCl. Then, the mixture was incubated at 90°C for 10 min to terminate the reaction. After centrifugation (12,000 ×g, 4°C) for 10 min, the supernatant of the hydrolysate was collected for test the ACE inhibitory activity.

### 2.3. Determination of ACE Inhibitory Activity

The ACE inhibitory activity was determined by Wang et al. method [27] with slight modifications. All samples were diluted to the same protein content (1.0 mg/mL), which was determined by Biuret assay method [28]. Sample solution (10 *μ*L) and ACE solution (50 units/mL, 30 *μ*L) were mixed together. After the mixture being preincubated at 37°C for 5 min, 50 *μ*L 7.6 mmol/L HHL substrate solution, which was solved in 50 mM sodium borate buffer and 6.8 mM NaCl at pH 8.3, was added. The mixture was incubated at 37°C for 25 min. The reaction was terminated after addition of 10 *μ*L of 20% trifluoroacetic acid (TFA). The solution was filtrated through 0.22 *μ*m membrane. The hippuric acid liberated by ACE was analyzed by reversed-phase high performance liquid chromatography (RP-HPLC) on an Inertsil ODS C_18_  (4.6 mm × 300 mm, 5 *μ*m) column. The mobile phase was 30% methanol, which contained 0.1% TFA and 0.05% acetic acid. The flow rate was 1.0 mL/min. The UV detection wavelength was 228 nm. The ACE inhibitory activity was obtained from peak area and expressed as percent. 

### 2.4. Choice of Protease

Five kinds of proteases were used for hydrolysis at their labeled optimum temperature and pH (shown in Table 1). And the protease was added at 50 U per mL mixture and the hydrolysis time was fixed at 4.0 h. Then ACE inhibitory activity of hydrolysate was determined. 

### 2.5. Uniform Design

A Uniform Design table of U_7_(7^4^) was applied to determine the optimum hydrolysis conditions for obtaining ACE inhibitory activity peptides from *Mytilus coruscus*. The combination effects of independent variables *X*_1_ (protease concentration, U/mL), *X*_2_ (hydrolysis time, h), *X*_3_ (hydrolysis pH), and *X*_4_ (hydrolysis temperature, °C) at 7 variation levels in the hydrolysis process were shown in Table 2. A total of 21 combinations (three replicates) for four factors were chosen according to Uniform Design table. The actual values were also shown in Table 2. The responses functions (*Y*) were ACE inhibitory activity. These values were related to the variables by a second-order polynomial (1) below:

(1) $$\begin{array}{c} Y=\beta_{0}+\sum_{i-1}^{m}\beta_{i}X_{i}+\sum_{i-1}^{m}\beta_{ii}X_{i}^{2}+\sum_{i<j}^{}\beta_{ij}X_{i}X_{j}, \end{array}$$

where *Y* is the predicted response. *X*_*i*_ and *X*_*j*_  are the independent variables. *β*_0_, *β*_*i*_, *β*_*ii*_, and *β*_*ij*_ were the regression coefficients. 

The significance was evaluated by Student's *t*-test. The actual values were compared with model predictions. The optimum hydrolysis conditions were verified by additional triplicate experiments under these conditions.

### 2.6. Amino Acid Composition Analysis

The amino acid analyses were conducted by the method of Noreen and Salim [29]. Briefly, the 10 mL of the sample was hydrolyzed under vacuum by addition of 10 mL concentrated HCl at 110°C for 24 h. When the free amino acids were analyzed, the sample did not hydrolyzed by HCl. Amino acids were analyzed in a Shimadzu HPLC system by separation in an ion-exchange column and post-column reaction with ninhydrin.

### 2.7. Statistical Analysis

Data were expressed as means ± standard deviation of triplicate. A probability value of *P* < 0.05 was considered significantly.

## 3. Results and Discussion

### 3.1. Choice of Protease

**Figure 1:**
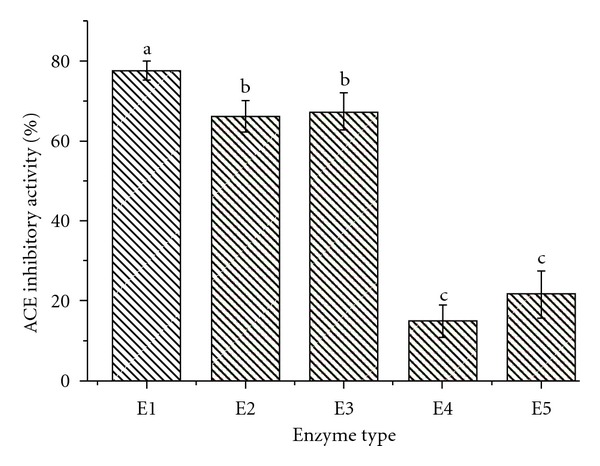
The ACE inhibitory activity of hydrolysates by five commercial proteases. The data was expressed as mean ± standard deviation of three determinations. Means sharing the same lowercase letter was not significantly different at *P* < 0.05.

ACE inhibitory peptides generally were short peptides and enzymatic hydrolysis of food derived protein was one of important measures to obtain ACE inhibitory peptides. A lot of ACE inhibitory peptides had been reported from food derived proteins hydrolysates. In this investigation, five kinds of commercial proteases, including three alkali proteases, one neutral protease, and one acid protease, were chosen to obtain ACE inhibitory peptides from *Mytilus coruscus*. The ACE inhibitory activity of various enzymatic hydrolysates was shown in Figure 1. From the results, it was shown that the hydrolysate produced by alkali protease E1 had the highest ACE inhibitory activity. In addition, alkali proteases (E1, E2 and E3) were more effective for hydrolysis *Mytilus coruscus* mussel protein to obtain ACE inhibitory peptides than other two proteases (E4 and E5). Therefore, alkali protease E1 was chosen to next experiments to optimize hydrolysis conditions for producing ACE inhibitory peptides from *Mytilus coruscus*.

### 3.2. Data Analysis of Uniform Design

A regression analysis was conducted to fit a mathematical model to the experimental data. The results of regression analysis were summarized (Table 2), and a regression equation was given in

(2) Y=0.1023+0.2626X1+0.1083X2+0.1100X3+0.1592X4 +0.0114X12+0.0937X22+0.1751X32+0.0020X42 −0.1404X1X2−0.0829X1X3−0.0314X1X4 +0.1561X1X3+0.2367X2X4+0.1960X3X4.

The statistical analysis indicated the predicted model was adequate, possessing significant *P*  value (*P* = 0.047 < 0.05) and satisfactory values of the regression coefficient *R* ^2^  (*R* ^2^ = 0.9712) for the response. The high regression coefficient make clear that the experimental values of the ACE inhibitory activity agreed with predicted values, which meant that the predicted model seemed to reasonably represent the observed values. The largest relative error of predicted value was less than 5% shown in Table 2. The significance was tested by Student's *t*-test and *P* value in Table 3. It was shown that temperature, pH, and protease added quantity affected significantly the ACE inhibitory activity of hydrolysates.

### 3.3. Verification Experiments

Then the optimum hydrolysis conditions of protease E1 and the prediction ACE inhibitory activity were obtained by (2). The optimum hydrolysis conditions of protease E1 were protease concentration (*X*_1_): 36.0 U/mL; hydrolysis time (*X*_2_): 2.7 h; hydrolysis pH (*X*_3_): 8.2; hydrolysis temperature (*X*_4_): 59.5°C. The predicted ACE inhibitory activity was 90.7% at optimum hydrolysis conditions. Under this optimum hydrolysis conditions, other three verification experiments were conducted and the average actual ACE inhibitory activity was 91.3%, which was in agreement with the predicted values of 90.7%. 

### 3.4. Amino Acid Composition

 The compositions of free amino acid and amino acid in peptides of the ACE inhibitory peptides from *Mytilus coruscus* were determined and the results were shown in Table 4. From the results, it was seen that the ACE inhibitory peptides solution had only a few free amino acid content, not equal with amino acid in peptides. The peptides, not amino acid, might contributed to high activity. The ACE inhibitory peptides had high percent of glutamic acid, taking 0.578 mmol/g, which could improve the breath ability of brain cell and be favorable to the expulsion of ammonia in brain and regulation of body metabolism, and these phases could impact the blood pressure directly. Also the ACE inhibitory peptides had high percent of lysine, leucine, glycine, and aspartic acid. These amino acids might play crucial role in the inhibitory activity. Cheung et al. [30] reported that dipeptides having hydrophobic amino acids such as valine (Val) and isoleucine (Ile) at the amino temerninus have higher ACE inhibitory activities. The amino acids, such as lysine, leucine, glycine, aspartic acid, and glutamic acid, were key constitutes with tall frequency appeared among many reported ACE inhibitory peptides [31–35].

## 4. Conclusions

Alkali protease was a good choice for hydrolyzing *Mytilus coruscus* protein for producing ACE inhibitory peptides. The factors, including protease concentration, hydrolysis time, hydrolysis pH, and temperature, affected the ACE inhibitory peptides of hydrolysates. Uniform Design was chosen to investigate the effects of preceding variables on ACE inhibitory activity. And the best hydrolysis conditions of alkali protease E1 optimized by Uniform Design were protease concentration of 36.0 U/mL, hydrolysis time of 2.7 hours, pH 8.2, temperature at 59.5°C. The optimal predicted ACE inhibitory activity of 90.7% was obtained at the optimum conditions. The experimental activity (91.3%) under optimized conditions was agreed closely with the predicted activity. The amino acid composition analysis of the ACE inhibitory peptides proved that it had high percent of lysine, leucine, glycine, aspartic acid, and glutamic acid. It was suggested that the ACE inhibitory peptides derived from *Mytilus coruscus* could be utilized to develop nutraceuticals and pharmaceuticals.

## Figures and Tables

**Table 1:** Hydrolysis conditions of the five proteases for producing ACE inhibitory peptides from *Mytilus coruscus. *

Protease	Temperature (°C)	pH	Time (h)
Protease E1	55	8.5	4.0
Protease E2	55	8.5	4.0
Protease E3	55	7.0	4.0
Protease E4	55	3.0	4.0
Protease E5	55	9.0	4.0

**Table 2:** Uniform Design with the observed responses and predicted values.

Treat	Variable levels				Experimental *Y*_*e*_	Predicted *Y *	LRE (%)
	*X*_1_	*X*_2_	*X*_3_	*X*_4_			
1	25	2	7.0	60	87.98 ± 3.14	88.08	4.8
2	50	4	8.5	55	77.21 ± 2.18	77.49	3.7
3	75	6	6.5	50	36.92 ± 1.91	36.83	5.0
4	100	1	8.0	45	26.54 ± 1.47	26.28	2.1
5	125	3	6.0	40	39.46 ± 2.31	39.02	3.6
6	150	5	7.5	35	58.13 ± 2.35	58.47	4.4
7	175	7	9.0	65	20.17 ± 1.17	20.46	3.2

*X*_1_: protease concentration, U/mL; *X*_2_: hydrolysis time, h; *X*_3_: hydrolysis pH; *X*_4_: hydrolysis temperature, °C; experimental *Y*_*e*_ was expressed as mean ± standard deviation of three determinations; LRE: largest relative error = 100 × |the largest or the lowest  *Y*_*e*_ − *Y*|/average *Y*_*e*_.

**Table 3:** Significance of regression coefficient for the ACE inhibitory activity.

Variables	Standard error	Computed *t* value	Significance level *P* value
*X*_1_	0.6374	5.2987	0.0501
*X*_2_	1.0897	0.9872	0.4619
*X*_3_	1.2345	5.6426	0.0478
*X*_4_	0.8766	7.2358	0.0342
*X*_1_ *X*_2_	−0.9234	4.4760	0.0323
*X*_1_ *X*_3_	−0.8768	3.9765	0.05926
*X*_1_ *X*_4_	−1.0236	7.0626	0.0355
*X*_2_ *X*_3_	0.7931	3.2617	0.0635
*X*_2_ *X*_4_	0.8942	6.2932	0.0433
*X*_3_ *X*_4_	0.6745	6.7869	0.0408
*X*_1_^2^	0.5679	5.4876	0.0496
*X*_2_^2^	0.9236	1.2381	0.3763
*X*_3_^2^	1.0111	4.2635	0.0543
*X*_4_^2^	0.8765	6.9367	0.0374

**Table 4:** Amino acid compositions of ACE inhibitory peptides from *Mytilus coruscus. *

Amino acid	Free amino acid content (mmol/g)	Amino acid in peptides (mmol/g)
Aspartic acid	0.009	0.449
Threonine	0.016	0.209
Serine	0.012	0.246
Glutamatic acid	0.013	0.578
Glycine	0.052	0.565
Alanine	0.027	0.558
Valine	0.009	0.155
Methionine	0.013	0.359
Isoleucine	0.018	0.157
Leucine	0.069	0.336
Tyrosine	0.000	0.089
Phenylalanine	0.020	0.146
Histidine	0.104	0.106
Lysine	0.068	0.419
Arginine	0.029	0.230
Cysteine	Not detected	Not detected
Proline	Not detected	Not detected
Tryphtophan	Not detected	Not detected
